# Complementarity and discriminatory power of genotype and otolith shape in describing the fine-scale population structure of an exploited fish, the common sole of the Eastern English Channel

**DOI:** 10.1371/journal.pone.0241429

**Published:** 2020-11-05

**Authors:** Marine Randon, Olivier Le Pape, Bruno Ernande, Kélig Mahé, Filip A. M. Volckaert, Eric J. Petit, Gilles Lassalle, Thomas Le Berre, Elodie Réveillac

**Affiliations:** 1 ESE, Ecologie et Santé des Ecosystèmes, Institut Agro - Agrocampus Ouest, INRAE, Rennes, France; 2 Statistical and Actuarial Science Department, Simon Fraser University, Burnaby, British Columbia, Canada; 3 Ifremer, Laboratory of Fisheries – Sclerochronology Centre, Boulogne-sur-Mer, France; 4 IIASA, Evolution and Ecology Program, Schlossplatz, Laxenburg, Austria; 5 Laboratory of Biodiversity and Evolutionary Genomics, KU Leuven, Leuven, Belgium; 6 UMR 7266 LIENSs, Littoral, Environnement et Sociétés, La Rochelle University - CNRS, La Rochelle, France; Institut de recherche pour le developpement, FRANCE

## Abstract

Marine organisms show population structure at a relatively fine spatial scale, even in open habitats. The tools commonly used to assess subtle patterns of connectivity have diverse levels of resolution and can complement each other to inform on population structure. We assessed and compared the discriminatory power of genetic markers and otolith shape to reveal the population structure on evolutionary and ecological time scales of the common sole (*Solea solea*), living in the Eastern English Channel (EEC) stock off France and the UK. First, we genotyped fish with Single Nucleotide Polymorphisms to assess population structure at an evolutionary scale. Then, we tested for spatial segregation of the subunits using otolith shape as an integrative tracer of life history. Finally, a supervised machine learning framework was applied to genotypes and otolith phenotypes to probabilistically assign adults to subunits and assess the discriminatory power of each approach. Low but significant genetic differentiation was found among subunits. Moreover, otolith shape appeared to vary spatially, suggesting spatial population structure at fine spatial scale. However, results of the supervised discriminant analyses failed to discriminate among subunits, especially for otolith shape. We suggest that the degree of population segregation may not be strong enough to allow for robust fish assignments. Finally, this study revealed a weak yet existing metapopulation structure of common sole at the fine spatial scale of the EEC based on genotypes and otolith shape, with one subunit being more isolated. Our study argues for the use of complementary tracers to investigate marine population structure.

## 1. Introduction

Recent advances suggest that, even in open habitats, populations of marine fish are commonly structured at relatively fine scales [1–3]. The degree of connectivity varies along a continuum of population segregation, from complete mixing (i.e. panmixia) to full isolation [4–6]. Somewhere in between, metapopulations display varying degrees of internal connectivity [1]. Mechanisms underlying the spatial structure of marine fish are (i) biophysical processes involved in egg and larval dispersal patterns [7,8] and (ii) post-larval (i.e. juvenile and adult) movements related to homing vs straying behavior and migration strategies [9]. The paradigm suggesting that larval dispersal acts as the main driver of population structure and connectivity [7] has been revised such that a significant contribution of adult-mediated dispersal is acknowledged [10]. Populations of marine resources experience many pressures among which habitat degradation and fragmentation, fishing exploitation and climate change [11]. In such a context, the resilience of marine species relies on their dispersal capability throughout their life cycle [12]. From a conservation point of view, assessing connectivity and spatial structure is crucial since isolation might put a population at risk and eventually lead to extinction if the isolated population is small and experiences external pressures. From a fisheries perspective, understanding population connectivity and spatial structure is a prerequisite to sustainable exploitation. In case of mismatch between biological population and harvest stock unit (i.e. the spatial unit used for assessment and management), overexploitation or even collapse might dramatically arise [13–16].

A wide range of methods exist to assess the structure and connectivity of marine fish populations [17,18]. Insights in population segregation are available from, among others, larval dispersal modelling [19,20], mark-recapture experiments [21,22] and natural tracers such as morphometry and meristics [23–26], microchemistry (e.g. [27,28]) and genetics [29,30]. These tools enable estimation of spatial population structure over ecological and evolutionary timescales [27]. Tracers covering an ecological time scale, like otolith-based tracers, inform about the population structure and connectivity throughout the fish life cycle. Genetic tracers provide information across generations at an evolutionary time scale. The choice of tracers is paramount since each has its own ecological interpretation, spatiotemporal resolution, discriminatory power and cost [27]. The comparison of tracers is advised since one single tracer may fail to detect population structure. If a tracer fails to detect heterogeneity, it might be because (i) the population is homogeneous, or (ii) because the spatiotemporal resolution of the tracer is not adapted to detect population structure, or finally (iii) because the discriminatory power of the tracer is too low. Among the broad panel of methods, genetics and otolith-based approaches are commonly used complementarily to resolve population structure and connectivity (e.g. [31–34]).

Genetic markers are well established tools used to inform on population structure at the evolutionary scale [29,30]. The main constraint of genetic markers is that limited exchanges of individuals suffice to maintain genetic homogeneity, hence failing to detect populations segregation over evolutionary time scales [35]. However, the power of genetic markers to detect subtle population differentiation is consistently increasing [36–38] and these markers now have the potential to detect fine-scale structure [39]. Especially, Single Nucleotide Polymorphisms (SNPs) are abundant and widespread changes in single nucleotides at loci situated in coding or non-coding regions of the genome [40]. SNPs are well adapted to detect weak genetic structuring at medium to fine spatial scales (e.g. [41–43]). However, failure to detect fine-scale population structure from genetic information is still a relatively common situation [35] and the use of complementary tracers is advised [44].

Otolith shape is a proven morphometric tracer suited to detect spatial population structure throughout the fish life cycle [44]. An otolith is a small calcified structure located in the inner ear of the fish. It grows continuously and conservatively from the birth to the death of the fish following its somatic growth dynamics [45]. Otolith external shape integrates the whole history of fish growth and is thus influenced by numerous and potentially confounding factors such as ontogeny (i.e. developmental stage, age, total fish length and sex), genotype and environment (e.g. hydrology, depth, substrate and diet composition; [46–48]). By focusing on individuals of the same cohort, length or sex, the ontogenetic influence is limited and spatial variations of otolith shape might be related to residual genetic and/or environmental effects, suggesting population spatial structure at an ecological timescale [44]. Moreover, compared to other natural tracers, otolith shape is relatively cheap and easy to use in routine with a dedicated software. Consequently, otolith shape may suitably complement genetic analyses to capture the various scales at which dispersal processes happen [49,50]. Comparing tracers that integrate information at the ecological and evolutionary time scales may allow to detect spatial population structure and its stability over time [51].

**Fig 1:**
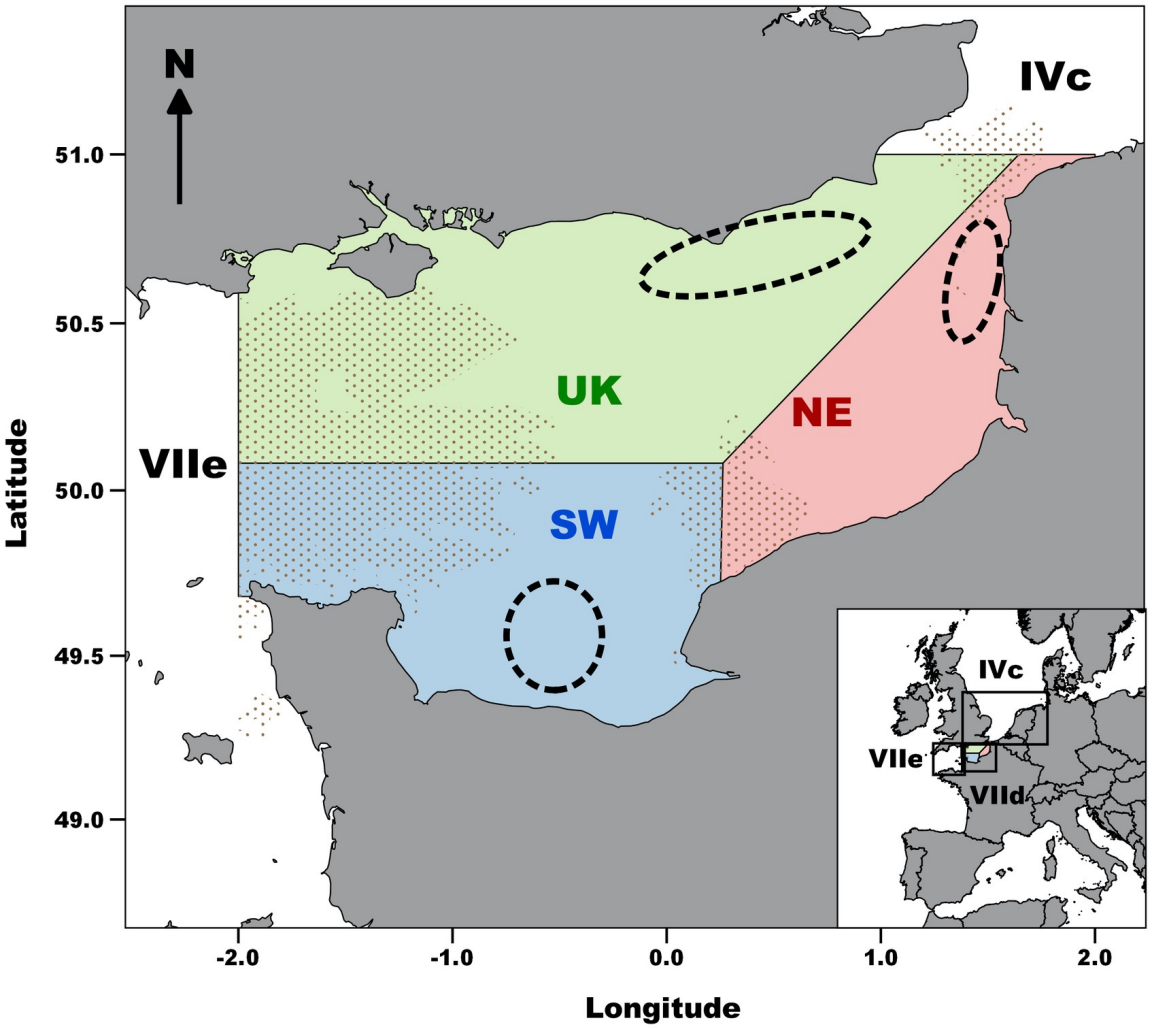
Map of the sampling sites of common sole in the three putative subunits (SW, NE and UK) of the Eastern English Channel stock (ICES area VIId). Dashed black ellipses show the sampling location of adults in the spawning grounds. Shading refers to rocky reefs.

The common sole (*Solea solea* (Linnaeus, 1758), Soleidae, Actinopterygii) of the Eastern English Channel stock (EEC; ICES division VIId; Fig 1) is a species of large economic interest that has been overexploited over the last decades [52]. This flatfish reproduces in early spring on spawning grounds off France and the UK. After hatching, larvae drift with currents towards shallow coastal nursery grounds where individual metamorphose [53]. Juvenile sole grow for about two years in coastal nursery grounds before joining the adult stock in deeper waters [54]. The internal structure of this stock has been questioned [55–57]. Biophysical modelling has suggested low larval connectivity [53] and high juvenile sedentariness has been evidenced from various approaches [58]. Based on the EEC underwater topography and the results of the biophysical modelling [53], a functioning in three subunits have been hypothesized (Fig 1). Life history traits at the population scale supported the spatial structure in three putative subunits ([59, 60]; Fig 1) and mark-recapture experiments estimated low exchanges between these subunits ([22]; Fig 1). However, the population structure has not been investigated yet at the individual level, nor a potential genetic differentiation. This study thus aimed to compare the discriminatory power of genetic and otolith shape analyses and assess their complementarity to describe the common sole population structure in the EEC. We first analyzed genetic structure over an evolutionary timescale with SNP genotypes. Then, we assessed population spatial structure over the lifespan using the phenotypic patterns of otolith shape. Finally, a supervised machine learning framework was applied on genetic markers and otolith shape descriptors to assess their respective discriminatory powers.

## 2. Material and methods

### 2.1. Ethic statement

In accordance with European Commission recommendation 2007/526/EC, on revised guidelines for the accommodation and care of animals used for experimental and other scientific purposes, fish sampling in the wild without experimental handling did not require an ethical agreement. Fish were caught with beam trawls or nets on professional fishing vessels or during a scientific survey. After being caught, fish were immediately immersed in ice to be sacrificed by hypothermia. The present field study did not involve endangered or protected species.

### 2.2. Sample collection

For the genetic analysis, a total of N_g_ = 215 (Table 1) adult common sole was sampled on the spawning grounds in French, off the rivers Seine (SW) and Somme (NE), and UK waters (Fig 1) in April and May 2017 and 2018 from commercial fishing vessels during tagging experiments [22]. An exception was made in July 2018, when individuals from the English part of the EEC were collected during the UK Beam Trawl Survey. Each fish total length was measured (in cm) and sex was determined by visual inspection of the gonads. A caudal fin clip was sampled and stored in pure ethanol for genetic analysis. For each fish, paired sagittal otoliths were removed and photographed. The right otolith was then used for age determination.

**Table 1:** Number of adult sole sampled within each subunit of the Eastern English Channel (SW, NE and UK subunits) used for genetic (Ng, split in Ng_2017_ and Ng_2018_) and otolith shape (Ns, split in Ns_2016_, Ns_2017_ and Ns_2018_) analyses.

	Ng_2017_	Ng_2018_	Ns_2016_	Ns_2017_	Ns_2018_
SW	47	41	354	128	17
NE	31	42	64	30	73
UK	42	12	12	80	21
EEC	120	95	430	238	111
Total	**N**_**g**_ **= 215**		**N**_**s**_ **= 779**		

The otolith sample size collected during tagging experiments and surveys appeared too low to ensure a reliable discriminatory power for the otolith shape analysis [27]. Otoliths collected from fish markets between 2016 and 2018 were thus added to increase the discriminatory power and resolution of the spatiotemporal analysis. A total of N_s_ = 779 were available for otolith shape analysis (Table 1).

A more precise description of the fish sampled for both genetic and otolith shape analyses is provided on S2 Appendix (**Table S2.1.**).

### 2.3. Genetic analysis

#### Construction of genomic libraries and bioinformatics

SNP markers were identified using double digest restriction-site associated DNA (ddRAD) sequencing on 215 adult sole (Table 1) [61]. DNA was extracted from fin clips [62]. Two separate libraries were built (i.e. samples of year 2017 / 2018) based on the protocol of [63] with the restriction enzymes *SbfI* and *SphI*. After enzymatic digestion and adapter ligation, sequences were size-selected (320–590 bp) and PCR amplified (16 cycles). Fragments between 300 and 600 bp were selected and libraries were sequenced paired-end on an Illumina HiSeq 2500 platform (Genomics Core, KU Leuven, Belgium).

*De novo* assembly was performed with the *dDocent* variant calling pipeline after demultiplexing [64]. More details about the *de novo* assembly and SNP calling are available in S1 Appendix. Because of stochasticity in generating RAD fragments [65,66], only 12 loci were left when combining samples from 2017 and 2018 during the SNP calling process. Consequently, the samples from 2017 and 2018 were analyzed separately.

After demultiplexing the 2017 library, 421 390 451 reads were available; 20 995 bi-allelic SNPs were retained through SNP calling. A comparable number of 234 348 163 reads was obtained from the 2018 library, resulting in 67 169 bi-allelic SNPs. These SNPs were filtered following criteria of allelic depth, allelic balance, allelic frequency, occurrence over all individuals, minimum heterozygosity threshold, Hardy-Weinberg Equilibrium (HWE) and threshold of linkage disequilibrium (LD). Information on these filters is provided in S1 Appendix. In 2017, 2 902 SNPs were retained for 120 individuals after SNP filtering. In 2018, 435 SNPs were retained for 95 fish.

#### Statistical analyses

Global and pairwise F_ST_ values [67] were evaluated using the *hierfstat* R package [68]. Significance of pairwise F_ST_ tests was computed by bootstrap (1000 permutations) and resulted in 95% interval credibility (i.e. 95% CI). A Discriminant Analysis of Principal Component (DAPC) was computed with the *adegenet* R package [69] for the 2017 and 2018 data sets separately. The number of PCs retained for the DAPC was assessed by the DAPC cross-validation procedure using the *xvalDapc* function of the *adegenet* R package. This procedure randomly leaves out a certain percentage of the data, runs DAPC, and then assesses if the data that was left out is correctly assigned into the population. Here, 90% of the whole data set composed the baseline and the remaining 10% individuals were assigned. The maximum number of PCs was set to 30 and 1000 replicates were computed.

### 2.4. Otolith shape analysis

After cleaning, pairs of otoliths were scanned with the sulcus side facing upward under reflected light at high resolution (3200 dpi). Individual images were extracted with the *TNPC 7* software (www.tnpc.fr).

#### Fourier analysis

The elliptical Fourier descriptors are among the most powerful methods based on otolith shape to discriminate among fish populations (e.g. [25,26,70,71]). This approach consists of extracting shape parameters from Fourier harmonics and investigating spatial differences in these parameters. The first 99 elliptical Fourier harmonics of each otolith were extracted from the scanned image via *TNPC 7* software. Normalization with respect to the first harmonic ensured the invariance of harmonics from the otolith size, rotation and starting point of contour description. Each harmonic *k*, described by 4 coefficients *a*_*k*_, *b*_*k*_, *c*_*k*_ and *d*_*k*_, yielded 392 shape descriptors (98 harmonics with 4 coefficients per harmonic). To reduce the number of descriptors, the number of harmonics ***n***_**j**_ of each otolith j was adjusted such that the contour was reconstructed with a precision of 99.9% (i.e., the proportion of variance in contour coordinates accounted for by the harmonics) as measured by the cumulative Fourier power **F** (Eq 1):

$$F(n_{\text{j}})=\sum_{k=1}^{n_{\text{j}}}\frac{a_{\text{k}}{}^{2}+b_{\text{k}}{}^{2}+c_{\text{k}}{}^{2}+d_{\text{k}}{}^{2}}{2}=99.9\%$$ (1)

The maximum number of harmonics *n* = max(*n*_*j*_) across all otoliths was then used to describe their contour to ensure a precision of at least 99.9% for each of them.

The number of elliptical Fourier descriptors was further reduced using a principal component analysis (PCA) with the *prcomp* function of the *stats* R package. The number of principal components (PC) was then chosen so that 99% of the variance was explained. The matrix of chosen principal components (S) thus represents the otolith shape matrix.

The differences in otolith shape between subunits was visualized using the mean otolith shape of each subunit formed by the outline reverse Fourier transform of the first *n* = max(*n*_*j*_) normalized harmonics. This visualization gave a first insight into spatial otolith shape variations.

Then, spatial (*Subunit*), total fish length (*Length*), otolith side (left or right, *Side*), sex (*Sex*) and sampling year (*Year*) effects were tested on the otolith shape matrix (S) using a redundancy analysis (RDA) with the *rda* function of the *vegan* R package [72] (Eq 2):

(S)~Subunit+Length+Side+Sex+Year (2)

Permutation tests using the *anova*.*cca* function assessed the relative influence of each variable on the shape matrix. This analysis informed on the strength of ontogenetic and spatial otolith shape variations.

#### Shape indices

In addition to Fourier analysis, otolith length *L*_*0*_ (i.e. the longest distance along the antero-posterior axis), width *l*_*0*_ (i.e. the longest distance along the ventro-dorsal axis), perimeter *P*_*0*_ and area *A*_*0*_, were measured to calculate shape indices [73] (Table 2).

**Table 2:** Shape indices as functions of otolith size measures [73].

Shape indices	Formulae
Ellipticity	*(L*_*0*_*-l*_*0*_*)/(L*_*0*_*+l*_*0*_*)*
Circularity	*P*_*0*_^*2*^*/A*_*0*_
Rectangularity	*A*_*0*_*/(L*_*0*_*×l*_*0*_*)*
Roundness	*(4A*_*0*_*)/(πL*_*0*_^*2*^*)*
Form coefficient	*(4πA*_*0*_*)/P*_*0*_^*2*^

*L*_*0*_, *l*_*0*_, *P*_*0*_ and *A*_*0*_ are the length, width, perimeter and area of otoliths, respectively.

Redundancy between shape indices was tested using the Pearson correlation test. Circularity and form coefficient (r = -0.99, p < 0.001) and ellipticity and roundness (r = -0.89, p < 0.001) were negatively correlated. Each shape index was kept in further analyses because they were not correlated with more than one index.

Spatial (*Subunit*), total fish length (*Length*), otolith side (left or right, *Side*), sex (*Sex*) and sampling year (*Year*) effects were tested on each shape index (*SI*) using a linear model with Gaussian error (Eq 3):

SI~Subunit+Length+Side+Sex+Year+ε (3)

The strength of each effect on shape indices was investigated using a type III *Anova* with the *car* R package. *Post hoc* tests with the *HSD*.*test* function of the *agricolae* R package [74] highlighted significant pairwise differences of shape indices between subunits.

### 2.5. Discriminatory power of genetic and otolith shape approaches

The discriminatory power of genetic and otolith shape approaches was investigated using the *assignPOP* R package. This package allows to analyze genetic, non-genetic and integrated data and is thus particularly suited to compare the discriminatory powers of genetic and otolith shape tracers. The package provides a machine learning framework whose principle is to assign individuals from different source populations by dividing the entire data set into training (i.e. baseline) and test data sets (i.e. unknown individuals) and building a machine learning classification function [75]. The predictive model is then applied to all unknown individuals (i.e. individuals that are not in the baseline) to assign them to their population of origin probabilistically. In practice, we applied a K-fold cross-validation procedure. The K-fold cross-validation method divided the whole data set in K subsets that were alternatively used as a training (i.e. baseline) or testing dataset (i.e. the remaining individuals). In our case, best accuracies were obtained by dividing each subunit into K = 3 groups. This procedure prevents from unbalanced training data sets among source populations [75]. Assignments of fish corresponded to the highest membership probability across the tests. An individual was correctly assigned if the predicted membership corresponded to the sampling subunit. Here, the predictive model was built using the Linear Discriminant Analysis (LDA) of the *MASS* R package [76]. The *assign*.*matrix* function of the *assignPOP* R package was used to compute a pairwise assignment matrix with mean and standard deviation of assignment accuracies across all assignment tests for each data type (i.e. genetic and otolith shape) and sampling year (i.e. 2017 and 2018). This method was used as a direct comparison of the discriminatory power of genetic and otolith shape approaches.

Prior to the assignment analyses, genetic and otolith shape data sets were pre-computed. Regarding the genetic approach, all loci were used to build the predictive model. Before the LDA was computed, a PCA was automatically applied on the genetic data for reducing the dimensions. Individual genotypes of the 2017 and 2018 data sets were clustered independently, since libraries were built separately.

Regarding the otolith shape approach, the predictive model was built using the more discriminant otolith shape descriptors among Fourier and shape indices or both (see 3.2). The potentially confounding factors on otolith shape (i.e. length, sex and otolith side) were removed prior to the clustering analysis using the residuals of linear models that tested these confounding effects on each otolith shape descriptors. In order to compare genetic and otolith shape discriminatory power, the 2017 and 2018 otolith shape data sets were also clustered independently (the discriminatory power analysis for 2016 otolith samples is not presented).

## 3. Results

### 3.1. Genetic analysis

Weak global genetic structure was found in 2017 and 2018 with low yet significant F_ST_ values (Table 3). Pairwise F_ST_ comparisons between subunits revealed distinct genetic pools between the three subunits in 2017 and between the SW and NE subunits in 2018 (Table 3).

**Table 3:** Pairwise F_ST_ values between the three subunits SW, NE and UK and the corresponding 95% confidence interval CI (upper and lower limits).

Year	Spatial comparison	Lower limit of 95% CI	F_ST_ value	Upper limit of 95% CI
2017	SW/NE	0.0019	0.0031*	0.0043
	SW/UK	0.0030	0.0044*	0.0060
	UK/NE	0.0045	0.0058*	0.0074
	Global	0.0035	0.0045*	0.0063
2018	SW/NE	0.0004	0.0031*	0.0058
	SW/UK	-0.0037	0.0028	0.0108
	UK/NE	-0.0033	0.0024	0.0095
	Global	0.0008	0.0029*	0.0132

‘*’ indicate significant values.

**Fig 2:**
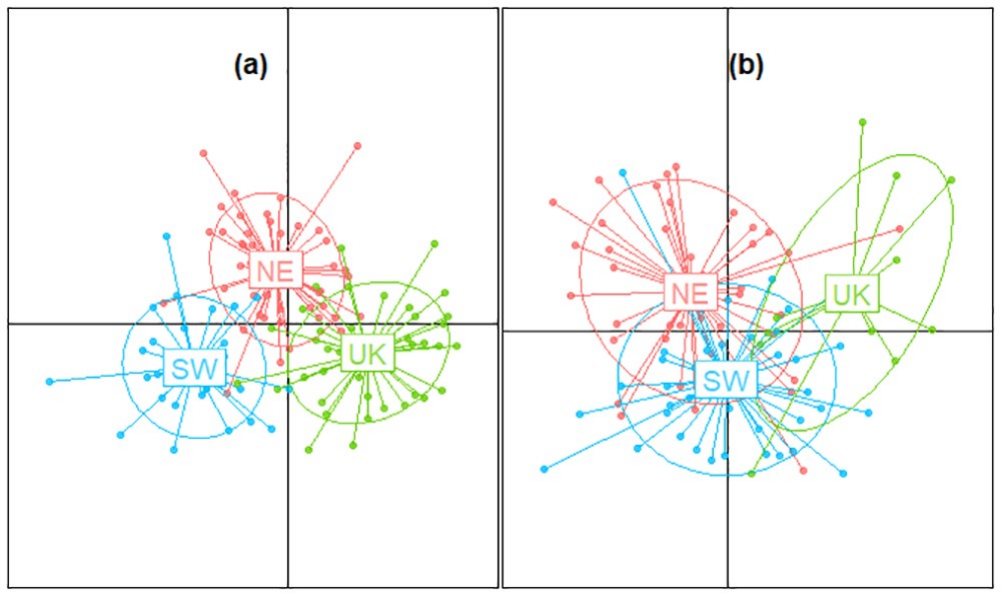
Plot of the Discriminant Analysis of Principal Components on the SNP genotypes of sole collected in 2017 (a) and 2018 (b).

These results were in line with the DAPC conducted on the 2017 and 2018 samples separately (Fig 2). 25 PCs and 20 PCs were retained for 2017 and 2018, respectively. The DAPC cross-validation procedure indicated that the mean successful assignment number was 71% in 2017 and 45% in 2018. Weak overlap between subunits was observed, especially for 2017 samples, supporting spatial genetic variation (Fig 2).

### 3.2. Otolith shape analysis

**Fig 3:**
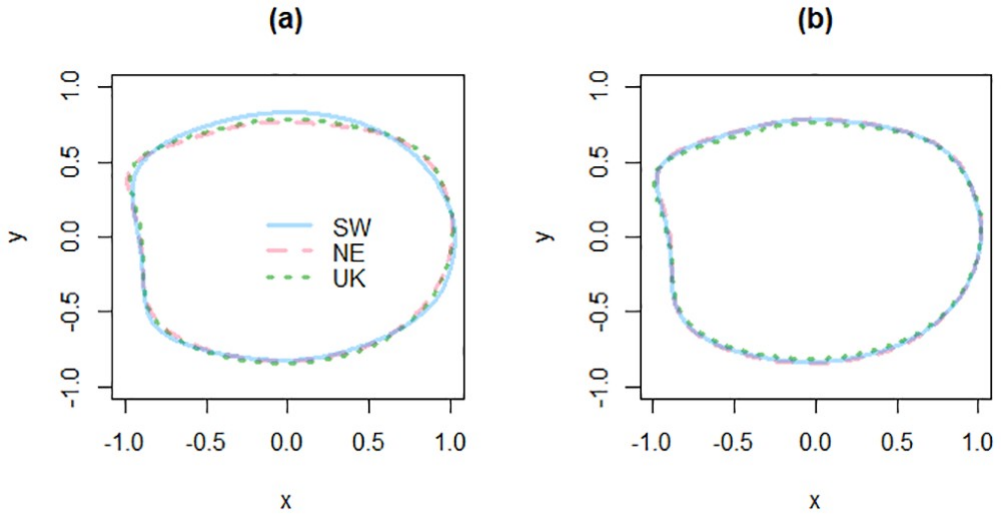
Mean otolith outline shapes formed by reverse Fourier transform of the outline using the first 28 harmonics for the left (a) and right (b) otoliths in the three subunits of the EEC. Values are centered and scaled.

Otoliths were reconstructed at 99.99% with 28 Fourier harmonics. The mean outline shapes of right and left otoliths were plotted to visualize the overlaps and variations between subunits (Fig 3). The spatial variations of the otolith mean shape appeared higher for the left otoliths, especially between the SW and the two other subunits (Fig 3a).

The dimension of the Fourier descriptors was reduced with a PCA that resulted in 33 principal components (PC) corresponding to 99% of total inertia. The redundancy analysis did not reveal spatial or temporal differences in Fourier descriptors (Table 4). Effects of total fish length, sex and otolith side were predominant.

**Table 4:** Results of the redundancy analysis and permutation test performed on the Fourier shape matrix composed of 33 principal components of otolith shape.

Variable	DF	F	p-value
Subunit	2	0.79	0.589
Length	1	7.48	0.001 ***
Side	1	128	0.001 ***
Sex	1	8.84	0.001 ***
Year	2	1.02	0.415

Statistical significance:

‘***’ P < 0.001.

In contrast, analysis of variance of shape indices highlighted significant spatial differences in form coefficient and circularity indices (Table 5).

**Table 5:** Results of the type III ANOVA performed on the otolith shape indices.

Shape index	Variable	DF	F	p-value
Ellipticity	Subunit	2	0.37	0.832
	Length	1	15.8	0.001 ***
	Side	1	81.3	0.001 ***
	Sex	1	12.3	0.001 ***
	Year	2	1.58	0.453
Circularity	Subunit	2	7.33	0.026 *
	Length	1	0.09	0.770
	Side	1	74.4	0.001 ***
	Sex	1	23.8	0.001 ***
	Year	2	7.87	0.020 *
Rectangularity	Subunit	2	0.31	0.857
	Length	1	2.21	0.137
	Side	1	0.97	0.325
	Sex	1	2.57	0.109
	Year	2	2.75	0.253
Roundness	Subunit	2	0.76	0.683
	Length	1	10.8	0.001 ***
	Side	1	74.6	0.001 ***
	Sex	1	7.89	0.001 ***
	Year	2	1.65	0.438
Form coefficient	Subunit	2	7.48	0.024 *
	Length	1	0.11	0.745
	Side	1	75.7	0.001 ***
	Sex	1	24.0	0.001 ***
	Year	2	7.48	0.024 *

Statistical significance:

‘*’ P < 0.5,

‘***’ P < 0.001.

More precisely *post hoc* tests indicated significant differences of circularity and form coefficient between the SW and NE subunits, with relation to the higher otolith metrics (i.e. otolith length, width, perimeter and area) in the SW subunits (S2 Appendix, **Table S2.2.**).

### 3.3. Discriminatory power of genetic and otolith shape approaches

#### Genetic approach

**Fig 4:**
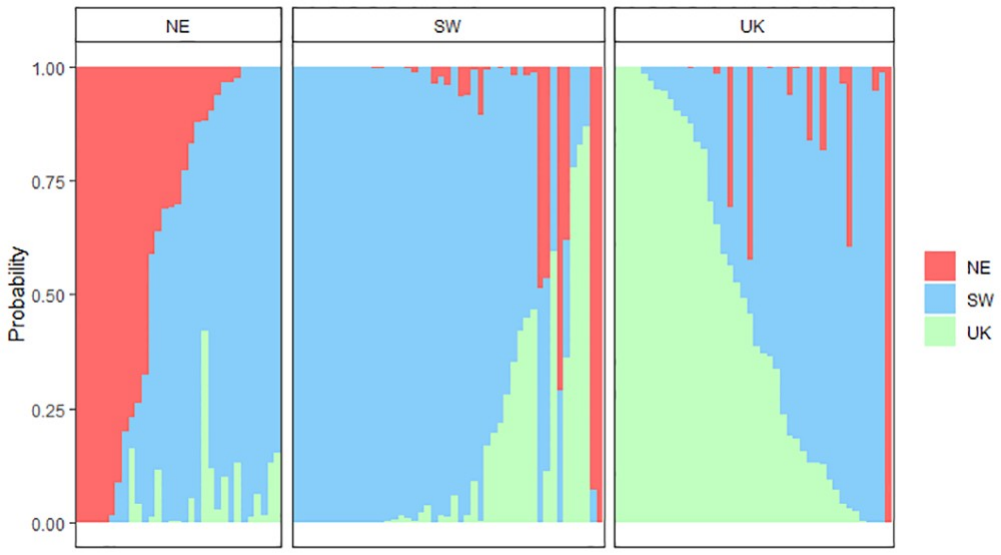
Membership probabilities of individuals in the three subunits based on the 2017 genotypes (Ng_2017_ = 120 with Ng_2017,SW_ = 47, Ng_2017,NE_ = 31 and Ng_2017,UK_ = 42). Each bar represents an individual sole. Individuals are ordered by increasing membership probabilities in each subunit. Panels correspond to the subunits where individuals were sampled. Probabilities were estimated using all loci (2902 SNPs) and K = 3 folds.

Genetic assignments revealed a weak yet significant genetic structure for 2017 samples with fish from the SW and NE subunits mainly assigned to the SW (Fig 4).

In 2017, across all the tests, mean self-assignment (i.e. assignment of individuals to their sampling subunit) was high in the SW (73%), moderate in the UK (60%) and low in the NE subunit (45%) (Table 6).

**Table 6:** Mean assignment percentages ± standard deviation of individuals across all assignment tests for each data type, sampling year and subunit. Grey cells indicate the subunit where individuals were mostly assigned.

			Estimated subunit of origin		
Data Type	Sampling Year	Sampling Subunit	SW	NE	UK
Genetic	2017	SW (Ng_2017,SW_ = 47)	0.73 ± 0.06	0.04 ± 0.04	0.23 ± 0.09
		NE (Ng_2017,NE_ = 31)	0.45 ± 0.08	0.38 ± 0.18	0.16 ± 0.12
		UK (Ng_2017,UK_ = 42)	0.38 ± 0.23	0.02 ± 0.04	0.60 ± 0.22
	2018	SW (Ng_2018,SW_ = 41)	0.73 ± 0.25	0.25 ± 0.22	0.02 ± 0.04
		NE (Ng_2018,NE_ = 42)	0.43 ± 0.19	0.57 ± 0.19	0 ±0
		UK (Ng_2018,UK_ = 12)	0.83 ± 0.14	0.17 ± 0.14	0 ± 0
Otolith shape	2017	SW (Ns_2017,SW_ = 128)	0.94 ± 0.05	0.01 ± 0.02	0.05 ± 0.05
		NE (Ns_2017,NE_ = 30)	0.74 ± 0.22	0 ± 0	0.26 ± 0.22
		UK (Ns_2017,UK_ = 80)	0.78 ± 0.10	0 ± 0	0.22 ± 010
	2018	SW (Ns_2018,SW_ = 17)	0 ± 0	0.97 ± 0.10	0.03 ± 0.10
		NE (Ns_2018,NE_ = 73)	0.05 ± 0.08	0.86 ± 0.14	0.09 ± 0.12
		UK (Ns_2018,UK_ = 21)	0.04 ± 0.09	0.92 ± 0.14	0.04 ± 0.09

**Fig 5:**
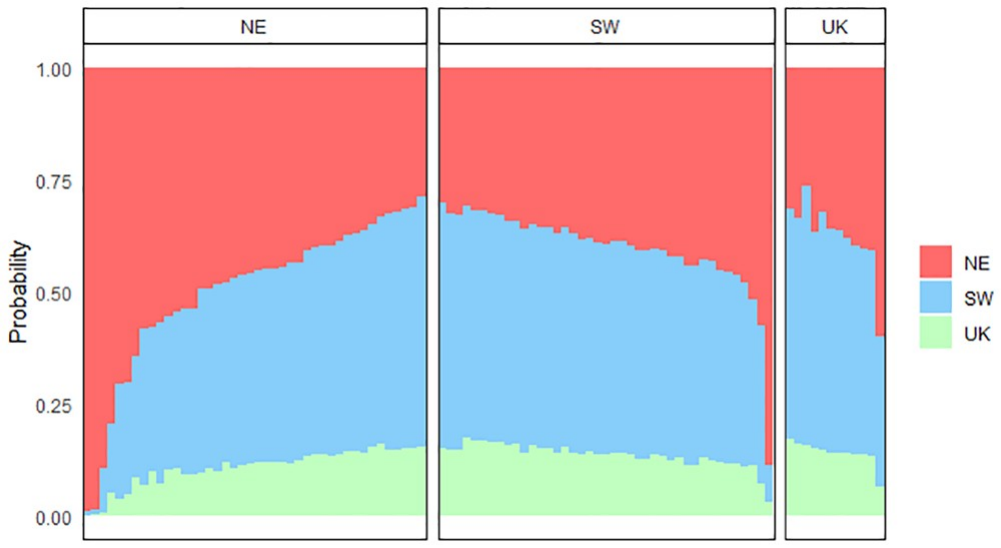
Membership probabilities of individuals in the three subunits based on the 2018 genotypes (Ng_2018_ = 95 with Ng_2018,SW_ = 41, Ng_2018,NE_ = 42 and Ng_2018,UK_ = 12). Each bar represents an individual sole. Individuals are ordered by increasing membership probabilities in each subunit. Panels correspond to the subunits where sole were sampled. Probabilities were estimated using all loci (435 SNPs) and K = 3 folds.

The 2018 genetic data revealed weaker population structure (Fig 5). Self-assignment was high in the SW (73%), moderate for the NE (57%) and null in the UK subunit (Table 6).

#### Otolith shape approach

**Fig 6:**
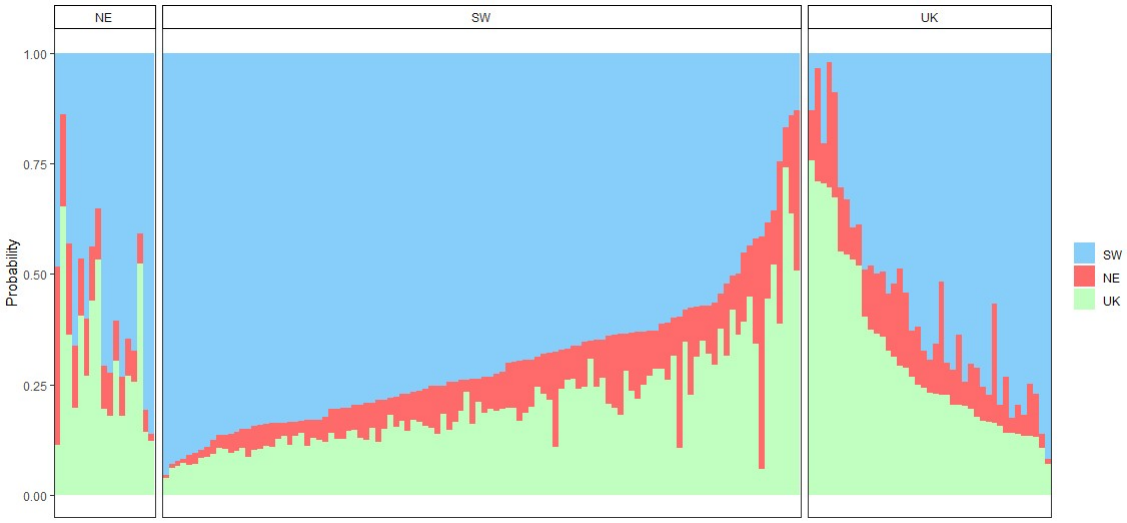
Membership probabilities of individuals in the three subunits based on the 2017 otolith shape data set (Ns_2017_ = 238 with Ns_2017,SW_ = 128, Ns_2017,NE_ = 30 and Ng_2017,UK_ = 80). Each bar represents an individual sole. Individuals are ordered by increasing membership probabilities in each subunit. Panels correspond to the subunits where sole were sampled. Probabilities were estimated using the residuals of a linear model that tested the length, sex and side effect on the otolith shape indices. Probabilities were estimated with K = 3 folds.

**Fig 7:**
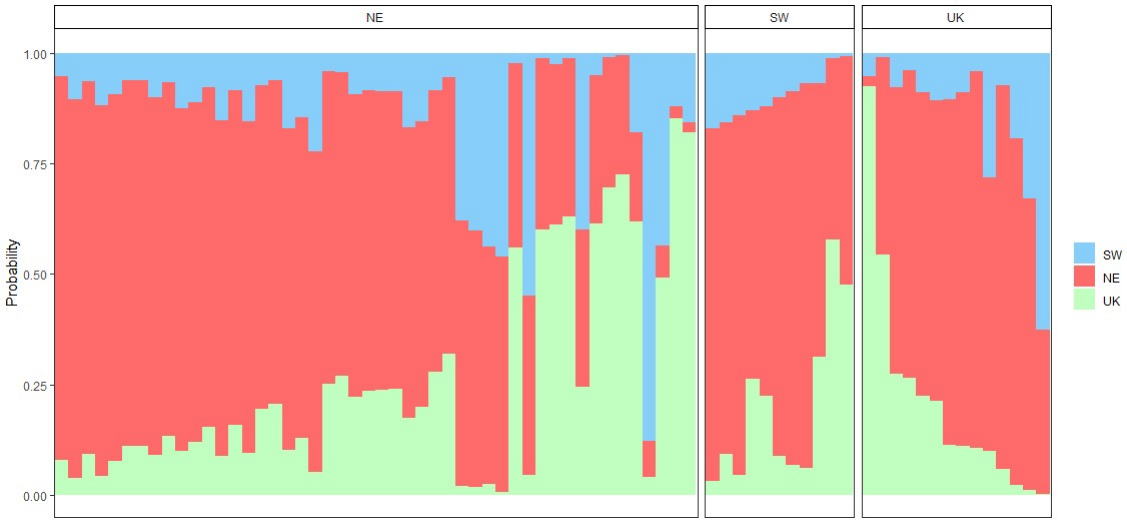
Membership probabilities of individuals in the three subunits based on the 2018 otolith shape data set (Ns_2018_ = 111 with Ns_2018,SW_ = 17, Ns_2018,NE_ = 73 and Ng_2018,UK_ = 21). Each bar represents an individual sole. Individuals are ordered by increasing membership probabilities in each subunit. Panels correspond to the subunits where sole were sampled. Probabilities were estimated using the residuals of a linear model that tested the length, sex and side effect on the otolith shape indices. Probabilities were estimated with K = 3 folds.

Otolith shape indices were selected for the discriminant analysis since Fourier descriptors failed to detect spatial variation in otolith shape (see 3.2). Compared to genetic assignments, otolith-based assignments revealed even lower discriminatory power for the 2017 (Fig 6) and 2018 (Fig 7) data sets. For the 2017 otolith shape data set, fish from each subunit were mainly assigned to the SW subunit, whereas in 2018, individuals were mostly assigned to the NE subunit (Table 6).

## 4. Discussion

In this study, we made progress with the understanding of the population structure of common sole in the EEC. The aim was to provide information on the population structure by comparing genetic and otolith shape analyses, and assessing their respective discriminatory power and complementarity. The genetic analysis highlighted low but significant differentiation between subunits, suggestive of a long-term weak population structure. The otolith-based approach provided a more unsettled signal of spatial structure. While Fourier descriptors did not detect spatial variation, straightforward shape indices suggested differences between two subunits. Finally, the comparison of the respective discriminatory power of genetic and otolith shape approaches revealed low self-assignment in subunits, especially for the otolith-based tracer.

### 4.1. Genetic differentiation at fine spatial scale

SNPs revealed spatial differentiation at a fine spatial scale between the SW, NE and UK subunits. Even if genetic differentiation (F_ST_ values) was low, pairwise F_ST_ values were significant for most comparisons, suggesting weak but significant isolation of the three proposed subunits over an evolutionary time scale. Moreover, low spatial overlapping of genotypes was highlighted by the DAPC, especially in 2017. The population structure of common sole at the scale of the North-East Atlantic Ocean based on microsatellites and mtDNA markers split in four groups. The North Sea and EEC group differentiated from the Bay of Biscay and to a lesser extent the Irish/Celtic Seas [77]. Using state-of-the-art SNPs, [78] confirmed a separation between the North Sea/English Channel population and the Bay of Biscay/Atlantic Iberian coast population. To the best of our knowledge, the present study was the first investigation of genetic differentiation on a fine spatial scale (i.e. < 200 km) of common sole in the North-East Atlantic Ocean.

The low F_ST_ values provided information on the degree of connection between subunits. However, such an analysis does not allow us to understand whether the connection is historical or whether the divergence between subunits is recent [79,80]. Thus, further investigations would be required to understand the process that generate the observed weak genetic structure.

We found that genetic spatial differentiation was less distinct in 2018 compared to 2017. Three explanations might be proposed for these temporal differences. First, the connection between subunits could have been stronger in 2018. This explanation is unlikely since a mark-recapture study has suggested low adult movement across the EEC over decades [22], which is in line with the spatial genetic pattern found in 2017. Second, the observed differences could be due to a library effect. According to empirical library comparisons and simulations, library preparation and sequencing result in variation in the rate of missing data rather than in population-level effects [65,66]. Third, the smaller sampling size and lower number of SNP markers in 2018 may have altered the power to detect population structure for this sampling year. This latter explanation is consistent with larger confidence intervals for F_ST_ in 2018 as compared to 2017 (Table 3).

### 4.2. Moderate spatial heterogeneity of otolith shape

We found no spatial variation of otolith shape using Fourier descriptors whereas the straightforward otolith shape indices suggested spatial differences of otolith shape between the SW and NE subunits. Various statistical methods have been developed to detect subtle variation in otolith shape. Fourier descriptors are particularly recommended in studies of spatial population structure and stock identification since they are considered as highly sensitive to variation in otolith shape [26,81]. Shape indices are basic otolith shape descriptors mainly used to supplement Fourier analysis [73]. Compared to Fourier descriptors, shape indices are expected to be less sensitive to subtle variation in otolith shape [82,83]. Moreover, Fourier descriptors and shape indices are supposed to be partially redundant information since Fourier descriptors are precise reconstructions of otolith outline whereas shape indices are considered as less integrative, yet accurate, metrics [84]. To our knowledge, not any study has previously presented spatially heterogeneous shape indices in a single analysis without significant spatial signal in Fourier descriptors. The high number of Fourier harmonics needed to reach the shape reconstruction threshold of 99% could be linked with these results. Too many subtle outline features compared to simpler yet perhaps more characteristic shape indices [26] might have led to draw different pictures of fish otolith (dis)similarities among spatial units. Although these contrasted results were unusual, they suggested that otolith shape of common sole varied spatially in the EEC.

Otolith shape is related to a complex combination of genetic, ontogenetic and environmental factors [47,85–87]. Here, the ontogenetic effect was neutralized considering fish length, year and sex effects together with side effect in the analysis. The results of otolith shape appeared congruent with the genetic findings, suggesting that the origin of otolith shape variations might be linked, to some extent, to genetic differentiation. Environmental factors such as water temperature and diet may also impact otolith shape through a change of growth [46,88]. However, in the EEC, environmental variables, and especially water temperature, are poorly contrasted [89]. The observed spatial pattern of otolith shape was in line with studies of life history traits at population scale: using the von Bertalanffy function to model the growth, [60] found that common sole from the SW presented higher asymptotic length than the two other subunits. Similarly, we found significant variation of otolith shape between the SW and the NE subunits. [60] proposed that the lasting signal of spatial pattern of growth might be due to contrasted fishing exploitation rate across the stock, with the SW subunit being the least exploited subunit. The theory behind this assumption is that fishing acts as a non-random genetic selection that favors individuals with early maturation and slow growth [90,91]. As a result, the lower exploitation rate in the SW subunit would favor larger fish. Therefore, the spatial variation of otolith shape was congruent with the result of long-term analysis of spatial pattern of growth for the common sole of the EEC. Therefore, a combination of genetic and environmental factors along with fishing pressure might contribute to the observed spatial pattern of otolith shape for the common sole of the Eastern English Channel.

Temporal variations of otolith shape were found for both Fourier descriptors and shape indices. Even if growth conditions vary between year (59), the spatial pattern of otolith shape was maintained from 2016 to 2018. Therefore, in this study, the strength of the spatial signal overrode the temporal pattern of otolith shape, suggesting a lasting spatial population structure.

### 4.3. Comparing the discriminatory power of tracers

Each tracer has its specific ecological meaning, but also its own resolution. Here the number of samples in each of the two approaches was based on previous knowledge of their resolution power [27]. This is the reason why additional samples were collected from fish markets to ensure a reliable discrimination power for otolith shape. Thus, sufficient samples were available for each analysis.

Our comparison of genetic and otolith shape discriminatory power demonstrated that the genetic approach outperformed otolith shape to discriminate among subunits in the EEC. However, both approaches showed low self-assignment percentages. In contrast to the F_ST_ and DAPC analyses, genetic assignment analysis suggested extremely weak spatial genetic structure within the population, with exchanges between subunits. This might be due to the low capability of the K-fold cross-validation method to accurately discriminate individuals from the three subunits using genetic data. There are a wide range of methods to assign or cluster individuals based on genetic data, each having advantages and limitations. For instance, other programs such as *GENECLASS2* [92] or *STRUCTURE* [93] propose to cluster individuals based on their genotype, but these methods tend to lesser perform when sampling is unbalanced between locations [94]. Considering that F_ST_ and DAPC results converged towards a genetic structure for the common sole, the results of the assignment analysis appeared weakly informative.

It was not surprising that the otolith shape discriminatory power was even lower since the strength of otolith shape spatial pattern was relatively weak, with a fewer number of discriminatory variables compared to SNP markers. Indeed, only two shape indices upon five (i.e. the circularity and form coefficient) allowed to detect spatial variations and those two shape indices were highly correlated. Moreover, the *assignPOP* framework is designed to assign both genetic and non-genetic data sets in an homogeneous manner [75]. This method allowed to compare the discriminatory power of genetic and otolith shape approaches. The *assignPOP* R package is supposed to limit the bias in assignment due to unbalanced sample size between sources [94]. We chose the K-fold cross-validation method to limit such a bias. However, whatever the tracer or year data sets used; assignment results suggested that individuals were mostly assigned into the subunits where sample size was the highest. Then, the unbalanced sample size between locations probably contributed to the low discriminatory power of genetic and otolith shape approaches. The low discriminatory powers observed might thus be due to the weak signal of spatial structure in the genetic and otolith shape data sets and to the unbalanced sampling between locations. The intensity of the spatial pattern of genetic and otolith shape was likely too small to assign individuals correctly. It logically underlined that the strength of the spatial pattern required for assignment tests is higher than for the simple detection of a spatial pattern.

### 4.4. Fine-scale population structure in the common sole of the Eastern English Channel evidenced from genetic and otolith-based approaches

The genotype and the otolith shape spatial variations were congruent and suggested a weak metapopulation (i.e. a set of subpopulations linked by dispersal; [95]) structure in three subunits, with a noticeable isolation of the SW subunit. These results were in line with former analyses of common sole population structure focusing on the adult stage. Estimation of von Bertalanffy growth parameters highlighted long-term differences between spatial subunits in the EEC [59,60]. In addition, density-at-age analysis suggested the isolation of the SW subunit from the rest of the stock [59,60]. Moreover, the results of a mark-recapture study demonstrated low dispersal across the EEC stock [22]. Therefore, it seems that the low connectivity observed at early life stages [53,58] is maintained at the adult stage and contributes to the weak metapopulation structure. Rocky reefs (Fig 1) and the deep central channel covered by gravels [96] are natural barriers for common sole in the EEC that could limit the exchanges of individuals [97], resulting in this metapopulation structure.

Finally, our genetic and otolith shape results confirmed previous analyses and suggested a misalignment between the common sole biological unit and EEC stock. The weak metapopulation structure proposed in this study should be considered in stock assessment and management to reach sustainable exploitation and long-term resilience of the metapopulation [30,98]. Ignoring even a weak metapopulation structure may lead to inaccurate estimate of population productivity and abundance and may bias the stock assessment and management [14,56], increasing the risk of overexploitation.

## Supporting information

S1 Appendix (DOCX)

S2 Appendix (DOCX)
