# Supplementary material for: Complementarity and discriminatory power of genotype and otolith shape in describing the fine-scale population structure of an exploited fish, the common sole of the Eastern English Channel
Source: PLoS One. 2020 Nov 5;15(11):e0241429. doi: 10.1371/journal.pone.0241429 (PMC7643961; doi:10.1371/journal.pone.0241429)
Supplement: S1 Appendix — (DOCX) [file pone.0241429.s001.docx]

Complementarity and discriminatory power of genotype and otolith shape in describing the fine-scale population structure of an exploited fish, the common sole of the Eastern English Channel

**RANDON** Marine^1*^, **LE PAPE** Olivier^1^, **ERNANDE** Bruno^2,3^, **MAHE** Kélig^2^, **VOLCKAERT** Filip A.M.^4^, **PETIT** Eric J.^1^, **LASSALLE** Gilles^1^, **LE BERRE** Thomas^1^ and **RÉVEILLAC** Elodie^1,5^

S1 - Appendix

- ***De novo* SNP assembly, SNP calling and filtering**

*De novo* detection of SNPs from ddRAD was chosen since no reference material was available for *Solea solea*. The assembly process was the same for each year of data, analyses separately, and all individuals of a sampling year were used with no distinction between cohorts. SNP calling was performed with the *dDocent* pipeline [1]. First, quality of reads was checked with *FastQC* [2] and forward and pair-end files were then demultiplexed with *process-radtags* of STACKS [3]. Quality trimming was performed using *Trimmomatic* [4]. The Paired-End (PE) RADseq algorithm of dDocent was used for de novo assembly. Forward reads were clustered using *CD-HIT* program [5]. Then, the *Rainbow* RadSeq assembly program recursively divided the clusters using reverse reads [6]. The percent similarity to clusters was set to 0.87. The quality-trimmed reads were mapped to the reference contigs with the *BWA-MEM* alignment algorithm [7]. We fixed the mapping match value to 2, the mapping mismatch value was set to 4, and the mapping gap opening penalty was 5. SNP calling was performed from the assembled haplotypes sequences with *FreeBaye*s variant detection software [8]. Finally, SNP were concatenated into a single variant call file (VCF) using *VCFtools* program [9].

Several filters were applied to reduce the number of SNPs and select relevant markers: minimum allelic depth = 4, allelic balance range = 0.25-0.75, minimum allele frequency = 0.05, exclusion of loci with missing data > 10% over all individuals. Loci with observed heterozygosity above 0.5 were removed. The Hardy-Weinberg equilibrium was estimated and distribution of missing data across spatial subunits and individuals and linkage disequilibrium were assessed using the *poppr* package [10]. Loci out of the Hardy-Weinberg equilibrium for more than 2/3 of individuals were removed. Also, loci in linkage disequilibrium above 0.7 were eliminated.

**References**

1. Puritz JB, Hollenbeck CM, Gold JR. dDocent: a RADseq, variant-calling pipeline designed for population genomics of non-model organisms. PeerJ. 2014;2: e431. doi:10.7717/peerj.431

2. Andrews. FastQC A Quality Control tool for High Throughput Sequence Data. In: Babraham Bioinformatics. 2010. Available: http://www.bioinformatics.babraham.ac.uk/projects/fastqc/

3. Catchen J, Hohenlohe PA, Bassham S, Amores A, Cresko WA. Stacks: an analysis tool set for population genomics. Mol Ecol. 2013;22: 3124–3140. doi:10.1111/mec.12354

4. Bolger AM, Lohse M, Usadel B. Trimmomatic: a flexible trimmer for Illumina sequence data. Bioinformatics. 2014;30: 2114–2120. doi:10.1093/bioinformatics/btu170

5. Fu L, Niu B, Zhu Z, Wu S, Li W. CD-HIT: accelerated for clustering the next-generation sequencing data. Bioinformatics. 2012;28: 3150–3152. doi:10.1093/bioinformatics/bts565

6. Chong Z, Ruan J, Wu C-I. Rainbow: an integrated tool for efficient clustering and assembling RAD-seq reads. Bioinformatics. 2012;28: 2732–2737. doi:10.1093/bioinformatics/bts482

7. Li H. Aligning sequence reads, clone sequences and assembly contigs with BWA-MEM. ArXiv13033997 Q-Bio. 2013. Available: http://arxiv.org/abs/1303.3997

8. Garrison E, Marth G. Haplotype-based variant detection from short-read sequencing. ArXiv12073907 Q-Bio. 2012. Available: http://arxiv.org/abs/1207.3907

9. Danecek P, Auton A, Abecasis G, Albers CA, Banks E, DePristo MA, et al. The variant call format and VCFtools. Bioinformatics. 2011;27: 2156–2158. doi:10.1093/bioinformatics/btr330

10. Kamvar Z, Tabima J, Grünwald N. Poppr: an R package for genetic analysis of populations with clonal, partially clonal, and/or sexual reproduction. 2014. Available: https://peerj.com/articles/281/
