# Supplementary material for: Complementarity and discriminatory power of genotype and otolith shape in describing the fine-scale population structure of an exploited fish, the common sole of the Eastern English Channel
Source: PLoS One. 2020 Nov 5;15(11):e0241429. doi: 10.1371/journal.pone.0241429 (PMC7643961; doi:10.1371/journal.pone.0241429)
Supplement: S2 Appendix — (DOCX) [file pone.0241429.s002.docx]

Complementarity and discriminatory power of genotype and otolith shape in describing the fine-scale population structure of an exploited fish, the common sole of the Eastern English Channel

**RANDON** Marine^1*^, **LE PAPE** Olivier^1^, **ERNANDE** Bruno^2,3^, **MAHE** Kélig^2^, **VOLCKAERT** Filip A.M.^4^, **PETIT** Eric J.^1^, **LASSALLE** Gilles^1^, **LE BERRE** Thomas^1^ and **RÉVEILLAC** Elodie^1,5^

S2 - Appendix

In this Appendix, we provided some complementary information on fish and otolith metrics.

In Table S2.1. we detailed the sampling.

**Table S2.1**. Proportion of females (F), males (M) and undetermined sex (U), mean otolith length, mean fish length and mean fish age for each data set, year and subunit. Sd indicates standard deviation.

| **Tracer** | **Year** | **Subunit** | **% F** | **% M** | **% U** | **Mean fish length ± sd (cm)** | **Mean fish age ± sd (year)** |
| --- | --- | --- | --- | --- | --- | --- | --- |
| Genetic | 2017 | SW | 67.4 | 8.70 | 23.9 | 28.1 ± 3.13 | 4.61 ± 1.24 |
|  |  | NE | 25.8 | 9.70 | 64.5 | 26.3 ± 2.07 | 4.07 ± 1.49 |
|  |  | UK | 42.9 | 4.76 | 51.8 | 27.9 ± 1.46 | 4.73 ± 1.35 |
|  | 2018 | SW | 41.7 | 33.3 | 25 | 30.0 ± 5.10 | 4.92 ± 2.07 |
|  |  | NE | 62.2 | 33.4 | 4.4 | 26.1 ± 8.83 | 4.24 ± 1.92 |
|  |  | UK | 42.1 | 55.2 | 2.70 | 25.1 ± 2.24 | 2.93 ± 1.11 |
| Otolith shape | 2016 | SW | 78.1 | 21.9 | 0 | 29.5 ± 3.22 | 3.50 ± 0.83 |
|  |  | NE | 75.1 | 24.9 | 0 | 29.1 ± 2.28 | 3.91 ± 1.21 |
|  |  | UK | 50.0 | 50.0 | 0 | 26.7 ± 2.77 | 4.17 ± 1.19 |
|  | 2017 | SW | 55.4 | 26.8 | 17.8 | 29.2 ± 3.09 | 4.37 ± 1.41 |
|  |  | NE | 65.9 | 18.8 | 15.3 | 26.8 ± 2.92 | 4.34 ± 1.38 |
|  |  | UK | 52.1 | 5.6 | 42.3 | 28.1 ± 1.46 | 4.63 ± 1.39 |
|  | 2018 | SW | 70.8 | 29.2 | 0 | 31.0 ± 5.49 | 5.54 ± 1.33 |
|  |  | NE | 53.8 | 30.8 | 15.4 | 26.8 ± 3.23 | 5.29 ± 1.66 |
|  |  | UK | 28.6 | 71.4 | 0 | 25.5 ± 2.60 | 4.86 ± 0.66 |

We also provided the distributions of the otolith shape indices per year and subunit (**Figure S2.1.**). Otolith length, width, perimeter and area (and fish total length in general) were higher in the SW compared to other subunits. It was likely due to the highest growth of fish in the SW subunit [1,2] since mean age of fish were sensibly the same in each case (except for the 2018 otolith data set where fish were a little bit older in average in the SW and NE subunits compared to the UK). Standardized to fish length, the spatial patterns of otolith shape metrics were maintained over the sampling year for each tracer. This result confirmed that, even if temporal variations in otolith shape existed, spatial variations were maintained.


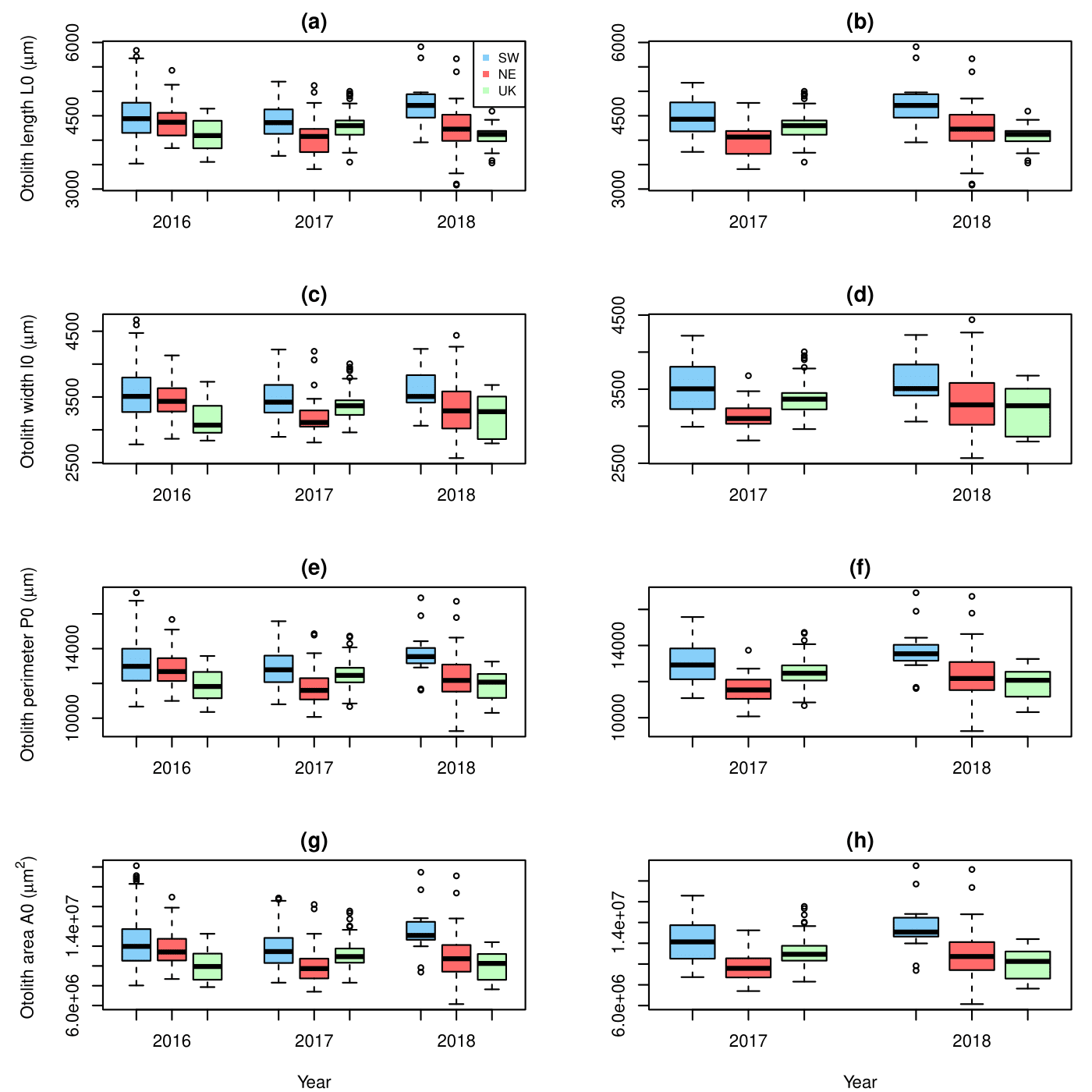


**Figure S2.1.** Boxplot of the otolith length (a, b), width (c, d), perimeter (e, f) and area (g, h) distributions per year and subunit for the otolith shape (left panels) and genetic (right panels) data sets.

**References**

1. Randon M, Réveillac E, Rivot E, Du Pontavice H, Le Pape O. Could we consider a single stock when spatial sub-units present lasting patterns in growth and asynchrony in cohort densities? A flatfish case study. J Sea Res. 2018;142: 91–100. doi:10.1016/j.seares.2018.09.012

2. Du Pontavice H, Randon M, Lehuta S, Vermard Y, Savina-Rolland M. Investigating spatial heterogeneity of von Bertalanffy growth parameters to inform the stock structuration of common sole, *Solea solea*, in the Eastern English Channel. Fish Res. 2018;207: 28–36. doi:10.1016/j.fishres.2018.05.009
